# Extended Right Hepatectomy for Giant Hepatic Hemangioma: A Case Report

**DOI:** 10.1155/cris/4016891

**Published:** 2026-06-30

**Authors:** Ana Gabriela Cabrera-Lizárraga, Omar Bermúdez-Ferro, Iaarah Montalvo-Gordon, Héctor Rendón-Dosal, Rubén R. Lozano-Salazar

**Affiliations:** ^1^ Escuela de Ciencias de la Salud, Universidad Marista de Mérida, Mérida, Yucatán, Mexico; ^2^ Hospital Christus Muguerza-Faro del Mayab, Mérida, Yucatán, Mexico; ^3^ Hospital Regional de Alta Especialidad de la Península de Yucatán-IMSS Bienestar, Mérida, Yucatán, Mexico

**Keywords:** case report, giant hepatic hemangioma, right hepatectomy, surgery

## Abstract

Liver hemangioma is the most common benign liver tumor, often discovered incidentally. Symptom development and complications underscore the need for surgical intervention. Managing hemangiomas larger than 20 cm is challenging because of the risk of perioperative complications. We report a case of a patient with compressive symptoms caused by a 20 cm liver hemangioma in the right hepatic lobe. An extended right hepatectomy was performed, yielding a favorable postoperative outcome. This case demonstrates that surgical treatment can be a safe and effective option for selected patients when performed at highly specialized centers, with outcomes comparable to those reported in the literature. It provides evidence that intraoperative surgical decision‐making is essential for an appropriate outcome in cases of giant symptomatic hemangioma.

## 1. Introduction

Hepatic hemangioma (HH) is the most common benign hepatic tumor. Most are smaller than 4 cm and are discovered incidentally. When they do not cause symptoms or complications, they typically do not require treatment or routine follow‐up. HH over 5 cm are classified as giant hemangiomas (GHs) and account for approximately 10% of cases [1, 2]. They typically localize in the right hepatic lobe [1]. Lesions over 20 cm are categorized as “extremely giant hemangiomas” (EGH) and are rarely reported [3]. Symptoms depend strictly on tumor size and location and include abdominal pain, gastric outlet obstruction, and compressive complications such as dyspnea, jaundice, lower‐limb edema, inferior vena cava compression, or Budd–Chiari syndrome [4, 5]. The management of EGH remains controversial. The size alone does not justify intervention. Clear surgical indications include pain, compressive symptoms, or potentially lethal complications [6]. Surgical options include formal resection or enucleation, determined by location, tumor morphology, and surgeon preference. Because managing lesions over 20 cm is highly complex, meticulous perioperative planning is critical. This case report describes the successful surgical management of an extremely giant HH in the right liver.

## 2. Case Presentation

A 46‐year‐old woman with no significant medical history presented to the emergency department with dyspnea and abdominal fullness that had developed over the past 10 days. On examination, the vital signs were normal. Abdominal assessment showed asymmetric enlargement, right hypochondrial tenderness, and a large palpable mass involving the epigastrium, right hypochondrium, and flank. Blood tests indicated hemoglobin 10.3 g/dL, leukocytes 3000/µL, platelets 189,000/µL, INR 0.9, and normal liver function tests. Contrast‐enhanced CT revealed an enlarged liver with heterogeneous parenchyma due to a lesion spanning all segments of the right lobe, measuring 23 cm × 15 cm × 20 cm (Figures 1 and 2). The right portal vein and its anterior branch appeared compressed by the mass. MRI showed a heterogeneously hyperintense mass on T2 and diffusion‐weighted images and hypointense on T1 images. Additionally, eight small, scattered lesions within the normal parenchyma, subsegmental atelectasis in the right lower lobe, and minimal pleural effusion were detected (Figure 3). The diagnosis was an extremely giant cavernous hemangioma of the right liver lobe. She was evaluated in a multidisciplinary session. We did not measure the functional liver remnant because the EGH occupied the entire right lobe, and the planned anatomic resection would not entail excessive sacrifice of healthy liver parenchyma. Compensatory preoperative hypertrophy of the residual liver had already occurred. The patient was considered fit for an anatomic liver resection. An extended right hemihepatectomy was performed via a rooftop incision. Mobilization was difficult because of the liver’s size and weight; the ligamentum teres was divided, and the falciform ligament was separated from the abdominal wall. However, we could not access the suprahepatic veins and the infrahepatic vena cava (IVC), nor could we incise the right coronary ligament; therefore, we changed the surgical plan to an anterior approach, beginning with vascular control of the IVC, followed by inflow control and dissection of the extrahepatic right portal pedicle under intraoperative ultrasound guidance. Given the compression of the hepatic hilum over the right portal pedicle on CT, the case was expected to be challenging. The hemangioma did not shrink as anticipated. Hepatic transection was performed with an ultrasonic scalpel, and three Pringle maneuvers were performed, each lasting 20 min. The operation lasted 600 min, with an estimated blood loss of 2400 mL. No vascular injuries occurred, and two units of blood were transfused. The patient was extubated at the end of surgery and transferred to the ICU without vasopressors. Postoperative blood tests showed hemoglobin 13 g/dL, leukocytes 17,300/µL, platelets 352,000/µL, INR 1.45, serum fibrinogen 167 mg/dL, ALT 40 U/L, AST 98 U/L, GGT 487 U/L, and glucose 135 mg/dL. A biliary fistula (Clavien‐Dindo grade II complication) was managed conservatively. The hospital stay was 9 days. Histopathology revealed a giant cavernous hemangioma weighing 2070 g and measuring 28 cm × 12.5 cm × 22 cm (Figure 4).

**Figure 1:**
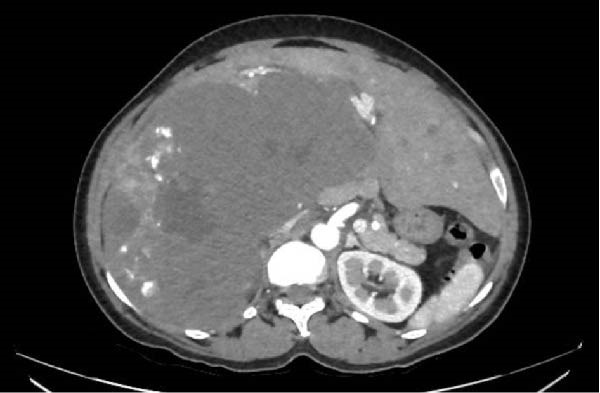
Contrast‐enhanced computed tomography (CECT) axial image. A well‐defined hyperdense lesion is visible in the right liver. Peripherally, late‐phase contrast uptake is characteristic of a giant cavernous hemangioma. The celiac trunk is displaced to the left due to the size of the liver.

**Figure 2:**
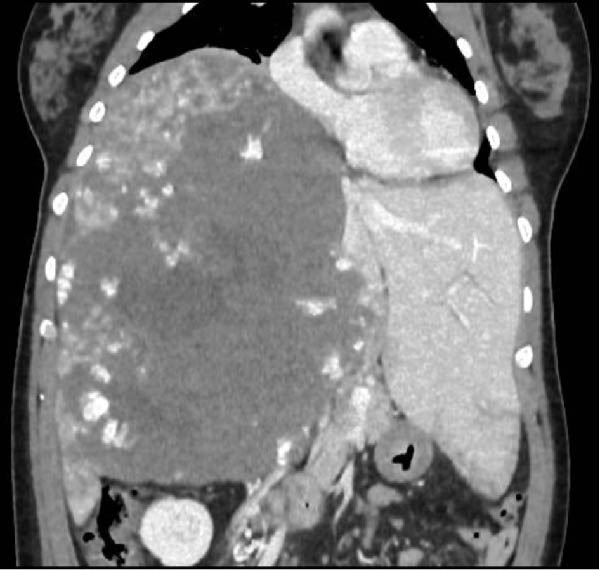
CECT coronal image. The late phase shows the typical progressive centripetal enhancement pattern of the lesion and its relation to nearby structures. Displacement of these structures is evident due to their size.

**Figure 3:**
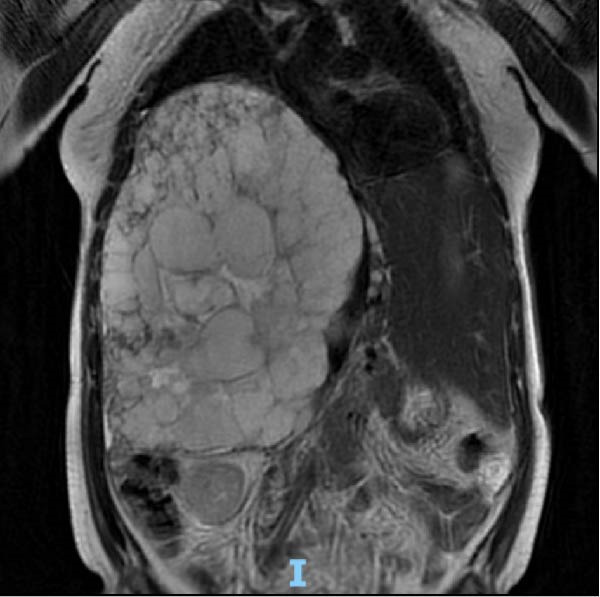
Abdominal magnetic resonance (MR) image, T1 sequence. A large multilobulated mass is visible in the right lobe of the liver, appearing heterogeneous and clearly hyperintense and displacing and compressing the hepatic and portal veins, as well as the vena cava, resulting in caudal displacement of the right kidney. The normal parenchyma of the left lobe of the liver is visible.

**Figure 4:**
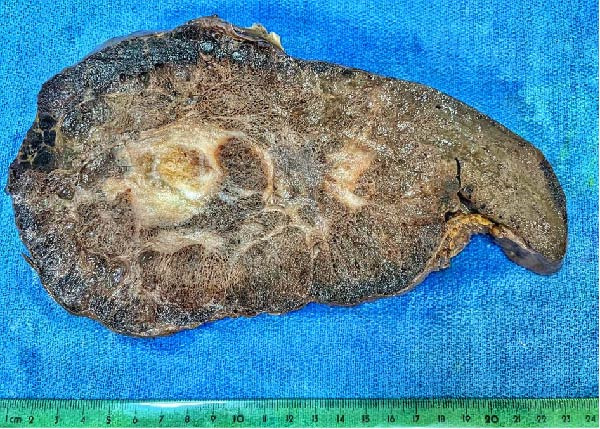
The macroscopic section of the surgical specimen.

## 3. Discussion

The anatomic complexity of an EGH and the surgical risk of a major hepatectomy make perioperative decision‐making challenging. Size alone does not justify intervention, but symptoms are related to the tumor size and location. Clear surgical indications include pain, compressive symptoms, or potentially lethal complications [6]. Operative treatment of EGH is described mostly in case reports, with only a few series [3]. Inflammatory complications may also occur, presenting as fever, pain, and bleeding (which can be intralesional or intraperitoneal, with the latter associated with hemangioma rupture) and may or may not involve consumptive coagulopathy [1]. Kasabach–Merritt syndrome is a specific entity that occurs mostly in the pediatric population with giant HH and is characterized by disseminated intravascular coagulation, thrombocytopenia, and microangiopathic hemolytic anemia. This syndrome is rare in adults [2, 6].

Conventional imaging studies have high sensitivity for diagnosing HH: MRI (100%), CT (98.3%), and ultrasound (96.9%), with specificities of 60%, 55%, and 86%, respectively. Cross‐sectional imaging is usually diagnostic. A biopsy is necessary only if the liver lesion is considered atypical and imaging does not yield a definitive diagnosis [7].

When surgery is indicated, options include an anatomic or nonanatomic segmentary resection or enucleation. Segmental resection is preferred for lesions in the left lobe or the posterior or lateral segments, especially when the lesion involves all affected segments or lobes. Surgical enucleation is an attractive option because it preserves healthy tissue, and the fibrous surgical plane facilitates dissection between the hemangioma and the adjacent liver parenchyma [8, 9]. Comparative studies show no differences in intraoperative bleeding, number of red blood cell units, operative time, vascular occlusion time, complications, or postoperative hospital stay [9, 10]. Small series suggest that enucleation is associated with fewer complications, possibly due to less bleeding [11].

Longer operative times reflect the complexity of this type of operation; our operative time was 10 h, which is above the average for an anatomic hepatic resection and reflects both the complexity of this case and our learning curve. It is similar to the time reported in a retrospective series from the largest referral hospitals in our country, with 57 patients with HH; of these, 32 had giant HH averaging 9 cm in diameter, and only nine were referred for surgery [6]. Operative time is shorter in patients with GH (>10 to <20 cm, median 12 cm, range 10–17 cm, 105 patients) than in those with EGH (>20 cm, median 24, range 20–50 cm, 36 patients) (3.5 ± 1.1 h vs. 5.3 ± 1.2 h, *p* < 0.001), as are blood loss (540.7 ± 468 vs. 1707 ± 1519 mL, *p* < 0.001), need for blood transfusion, and postoperative hospital stay [3]. There is considerable variability in operative times, primarily due to HH size and location, with reported values ranging from 40 to 600 min [9–13].

HH typically consists of variably sized, blood‐filled vascular channels lined by a single layer of endothelial cells, fed by the arterial circulation, with enormous vascular spaces, no cellular atypia, and interspersed with connective tissue septa [4]. Because of this, preoperative portal vein embolization or the ALPPS procedure (associating liver partition and portal vein ligation for staged hepatectomy) will not be effective strategies for increasing the remnant liver volume [14]. In an EGH occupying an entire hemiliver, especially the right lobe, little normal liver parenchyma remains on the affected side, and an anatomic resection will not result in excessive sacrifice of healthy liver parenchyma [10]. There is minimal or no risk of postoperative liver failure when most of the surgical specimen is replaced by the tumor lesion and the expected loss of functional liver is small, as compensatory preoperative hypertrophy of the residual liver has already occurred [15]. However, postoperative liver failure has been reported in a large retrospective series [9]. In our case, the hemangioma weighed more than 2000 g. Inflow occlusion, achieved with the Pringle maneuver or selective arterial clamping, can reduce the size of a hemangioma. When possible, vascular control at the level of the suprahepatic veins should be performed. Total vascular occlusion and extracorporeal circulation may be necessary in the presence of EGH, with a high risk of bleeding [12].

Factors associated with increased intraoperative bleeding and a longer postoperative stay include lesion size, location, relationship to vascular structures, prothrombin time, procedure duration, vascular occlusion, and the development of complications [8, 9]. The complication rate is high, around 40%, but most are minor (Clavien–Dindo [16] grade I‐II). The average intraoperative bleeding is 400 mL (range 50–10,000 mL), and one third of patients require transfusion. Mortality in large series is low, ranging from 0% to 5% [10, 14, 15].

Patients with EGH typically have a higher incidence of anemia, low platelet count, coagulation abnormalities, compression of vascular structures, and a higher risk of severe nonoperative complications, all of which increase surgical risk [3]; thus, surgical treatment has been considered even before the appearance of habitual symptoms. Recurrence or enlargement of residual small hemangiomas is rare; therefore, synchronous smaller lesions do not require resection. Liver transplantation is considered a salvage procedure for unresectable lesions or those that develop complications that cannot be managed in any other way, such as life‐threatening coagulopathies associated with Kasabach–Merritt syndrome [3, 12]. Some series and case reports have reported the feasibility of laparoscopic enucleation for GHs, aided by infrahepatic IVC clamping and the Pringle maneuver [17, 18]. Given the minimal risk of postoperative liver failure and the absence of a liver transplantation program in the southern part of our country, we feel safe performing complex hepatopancreatobiliary procedures. These patients should be evaluated at specialized centers; in an individualized approach, we must consider the lesion’s size, location, symptoms, and potential complications. Surgical resection remains the treatment of choice in most cases.

## Funding

This work received institutional funding from the Hospital Regional de Alta Especialidad de la Península de Yucatán (Program PP Q008).

## Conflicts of Interest

The authors declare no conflicts of interest.

## Data Availability

The data that support the findings of this study are available from the corresponding author upon reasonable request.
